# Low-Threshold Microlasers Based on Holographic Dual-Gratings

**DOI:** 10.3390/nano11061530

**Published:** 2021-06-09

**Authors:** Tianrui Zhai, Liang Han, Xiaojie Ma, Xiaolei Wang

**Affiliations:** Faculty of Science, College of Physics and Optoelectronics, Beijing University of Technology, Beijing 100124, China; hanliang@emails.bjut.edu.cn (L.H.); xiaojiema@emails.bjut.edu.cn (X.M.)

**Keywords:** microlasers, holographic, dual-gratings

## Abstract

Among the efforts to improve the performances of microlasers, optimization of the gain properties and cavity parameters of these lasers has attracted significant attention recently. Distributed feedback lasers, as one of the most promising candidate technologies for electrically pumped microlasers, can be combined with dual-gratings. This combination provides additional freedom for the design of the laser cavity. Here, a holographic dual-grating is designed to improve the distributed feedback laser performance. The holographic dual-grating laser consists of a colloidal quantum dot film with two parallel gratings, comprising first-order (210 nm) and second-order (420 nm) gratings that can be fabricated easily using a combination of spin coating and interference lithography. The feedback and the output from the cavity are controlled using the first-order grating and the second-order grating, respectively. Through careful design and analysis of the dual-grating, a balance is achieved between the feedback and the cavity output such that the lasing threshold based on the dual-grating is nearly half the threshold of conventional distributed feedback lasers. Additionally, the holographic dual-grating laser shows a high level of stability because of the high stability of the colloidal quantum dots against photobleaching.

## 1. Introduction

Different types of gain materials have been applied to microcavity lasers, including dyes, polymers, and colloidal quantum dots (CQDs) [1,2,3,4,5,6]. CQDs have demonstrated great potential for use as gain materials because of advantages that include their high photoluminescence quantum yields (PLQYs) and the low-cost and effective chemical manufacturing processes required [7]. The PLQY is more than 85%, and the full width at half maximum (FWHM) of the photoluminescence is approximately 30 nm. CQDs thus represent a great choice for use as gain materials in low-threshold distributed feedback (DFB) lasers or other microcavity lasers. Many methods can be used to fabricate DFB cavities and other microcavities, including self-organization [8,9], electron beam lithography [10,11,12], nanoimprinting [13,14,15], direct writing [16,17,18], and interference lithography [19,20,21]. Interference lithography provides a simple and low-cost method to fabricate dense periodic nanostructures rapidly over a large area. Among the efforts to improve the performance levels of microlasers, the optimization of the laser’s gain properties and cavity parameters has attracted significant attention recently [22,23,24,25]. Apart from the need for a better gain material, there is a need to optimize the cavity to decrease the threshold of DFB lasers. The laser threshold is strongly dependent on the loss and gain properties of the cavities. Here, optimization of both the output (loss) and the feedback (gain) for DFB lasers is performed by designing a special cavity.

In general, the DFB cavity contains a film composed of a gain material and a grating; in this cavity, the second-order diffraction of the grating leads to feedback, and the first-order diffraction of the grating leads to the output [26]. In this configuration, the first-order diffraction intensity is always higher than that of the second-order diffraction of the grating. Therefore, an excessive output is set up when compared with the feedback and results in a major loss and a high lasing threshold. The introduction of substructures to the regular DFB cavity can reduce the excessive output energy and also significantly improve the laser performance.

In this paper, we design a CQD-based microlaser based on a holographic dual-grating. The laser device consists of a CQD-based film that is used as a gain material and a dual-grating for use as a DFB structure, which is fabricated using interference lithography. The holographic dual-grating is formed by superposing a first-order grating with a second-order grating. After careful adjustment of the structural parameters, the thresholds of the developed dual-grating lasers are nearly half the thresholds of conventional DFB lasers.

## 2. Fabrication Methods

A photoresist (PR, AR-P3170, Strausberg, Germany) solution is spin-coated on a glass substrate (dimensions: 15 × 15 × 1 mm^3^) (Indium-Tin Oxide, ITO) at 2500 r/min for 30 s and is then heated for 1 min at 110 °C, forming a film that is approximately 180 nm thick. The holographic dual-grating structure, which consists of a first-order grating and a second-order grating, is fabricated in the PR film via interference lithography. The grating period (Λ) is determined using the following formula:

Λ = λ/(2sin(α))(1)

where λ is the wavelength of the laser used to perform interference lithography and 2α is the included angle between the two incident beams used to perform interference lithography.

**Figure 1:**
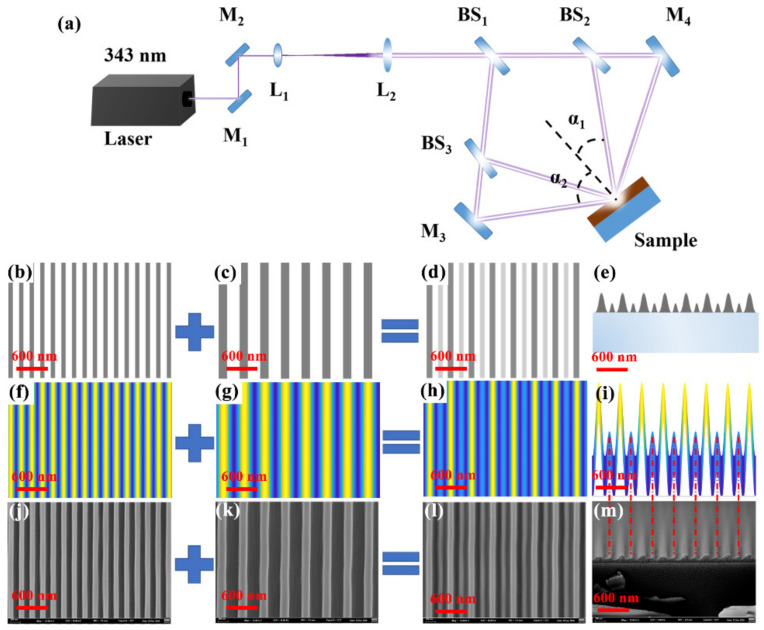
(**a**) Optical layout used for interference lithography. M_1_, M_2_, M_3_, and M_4_ represent reflectors. L_1_ and L_2_ represent lenses. BS_1_, BS_2_, and BS_3_ represent beam splitters. Angles 2α_1_ and 2α_2_ correspond to the included angles between the two interference beams. Schematics of (**b**) the first-order grating, (**c**) the second-order grating, and (**d**,**e**) the holographic dual-grating. (**f**–**i**) Simulated interference patterns corresponding to the structures shown in (**b**–**e**), respectively. (**j**–**m**) SEM images corresponding to the structures shown in (**b**–**e**), respectively.

The optical layout required for interference lithography is illustrated in Figure 1a. A 343 nm pulsed laser (FLARE NX, Coherent, Santa Clara, CA, USA) with 1 ns pulse duration, 3 mm spot size, and an adjustable pulse energy from 0 to 100 μJ at a 600 Hz repetition rate was used as the ultraviolet laser source. The first-order grating is fabricated using two-beam interference lithography, in which the included angle between the two incident beams is 2α_2_ (109.5°). The exposure and development time are 40 s and 8 s, respectively. A schematic, a simulated interference pattern, and a scanning electron microscopy (SEM, Hitachi S-4800, Hitachi, Tokyo, Japan) image of the first-order grating (210 nm) are shown in Figure 1b,f,j, respectively. Corresponding images for the fabricated second-order grating (420 nm), where the included angle between the two interference beams is 2α_1_ (48.2°), are shown in Figure 1c,g,k, respectively. The ridge width of the nanoscale grating is about 100 nm. The exposure and development time are 25 s and 9 s, respectively. After two exposures, the same images for the fabricated dual-gratings are shown in Figure 1d,h,l, respectively. The corresponding cross-sectional images of the holographic dual-grating are shown in Figure 1e,i,m, respectively. The interference patterns shown in Figure 1f–i were simulated using the commercial software MATLAB (R2014a, The Math Works, Natick, MA, USA). The bright and dark interference fringes in these patterns are indicated by the yellow and blue regions, respectively. All the exposure and development time are carefully optimized through a design of experiments.

**Figure 2:**
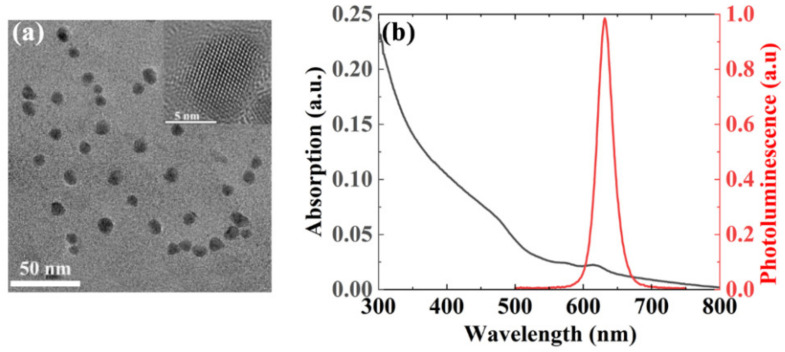
(**a**) TEM image of CQDs. The inset shows an HR-TEM image of the CQDs. (**b**) Absorption and photoluminescence spectra of the CQDs.

The transmission electron microscope (TEM) image (Measured by Beijing Beida Jubang Science & Technology Co., Ltd., Beijing, China) of the CQDs shown in Figure 2a indicates that the size of the nanocrystals is approximately 10 nm. The CQDs material is a typical nanomaterial that is usually employed as a gain medium. The inset shows a high-resolution TEM (HR-TEM) image of the CQDs, which indicates that the lattice constant is approximately 4 Å. The spectroscopic characterization results for these CQDs are shown in Figure 2b. The absorption and photoluminescence (PL) spectra measured by a spectrometer (Duetta, Horiba Instruments Inc., Irvine, CA, USA) are shown as the purple curve and the red curve, respectively. The PL peak is located at 630 nm, and the FWHM is approximately 30 nm, as shown in Figure 2b. The CQDs can be fabricated in a smooth film via spin coating, and the PLQY exceeds 85%.

A solution of CQDs is prepared by dissolving CQDs into toluene with a concentration of 40 mg/mL. Then, the toluene solution of CQDs is spin-coated on the grating structures at a speed of 1800 rpm/min for 30 s, forming a CQDs film around 200 nm. The refractive index of CQDs film is around 1.85.

## 3. Discussion

**Figure 3:**
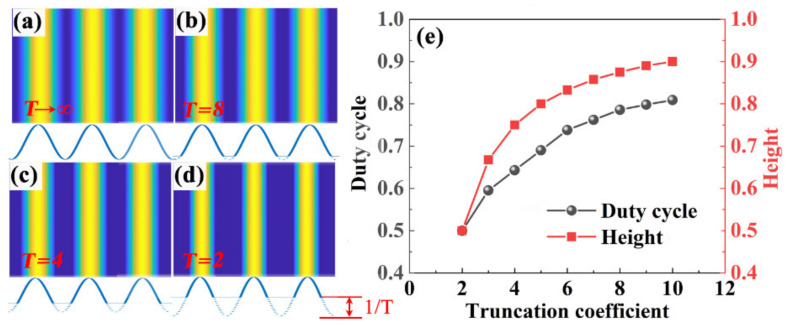
Simulated grating structures for different exposure times. The insets indicate the cross-sectional profiles of corresponding gratings. (**a**) T is ∞. (**b**) T is 10. (**c**) T is 4. (**d**) T is 2. (**e**) Duty cycle and height of the grating as a function of the truncation coefficient.

In the experiment, the duty cycle and height of the grating can be controlled directly by the exposure time Figure 3a–d shows the simulated patterns with different exposure times. The truncation coefficient (T) is introduced to indicate the degree of overexposure, which influences the profile of the grating. As shown in the insets in Figure 2a–d, the grating structure is truncated by a height of 1/T of the grating depth at the bottom. Obviously, the duty cycle and height of the grating decreases with a decreasing truncation coefficient, which can be controlled quantitatively by the degree of overexposure, as shown in Figure 2e.

According to laser theory, the threshold of the proposed laser based on the dual-grating structure depends on the gain and the loss in the cavity. For DFB lasers, the gain and loss characteristics of the cavity mainly originate from the feedback and the output, respectively. The feedback coefficient (*C_f_*) and the output coefficient (*C_0_*) for second-order DFB lasers can be defined as follows: [27,28].

(2) $$C_{f}=\frac{\sigma_{2}\Delta \varepsilon }{2\lambda_{0}^{2}K_{0}}{\int \left|\varphi \left(x\right)\right|}^{2}dx$$

(3) $$C_{0}=\frac{\sigma_{1}^{2}\Delta \varepsilon }{4\lambda_{0}^{4}K_{0}K_{x}}{\int \left|\exp\left(ixK_{x}\right)\varphi \left(x\right)\right|}^{2}dx$$

where *σ*_1_ and *σ*_2_ are the first- and second-order Fourier coefficients, respectively. In addition, Δ*ε* is the refractive index contrast of the grating, *λ_0_* is the wavelength in vacuum, *K_x_* is the propagation vector of the radiation and *K*_0_ is the grating Bragg vector. *φ*(*x*) is the transverse mode profile and exp(*ixK_x_*) describes the wave scattered in the x-direction. According to Equations (1) and (2), *C_f_* and *C_0_* can be controlled by adjusting the Fourier coefficients *σ*_2_ and *σ*_1_, respectively. Here, *C_f_* and *C_0_* can be regarded as the gain and the loss, respectively. In other words, the DFB laser threshold can be controlled by adjusting the Fourier orders *σ*_1_ and *σ*_2_ or their ratio *σ*_2_/*σ*_1_. A larger ratio corresponds to a lower threshold. Note that the Fourier coefficients are the spatial components that describe the height modulation of the grating.

**Figure 4:**
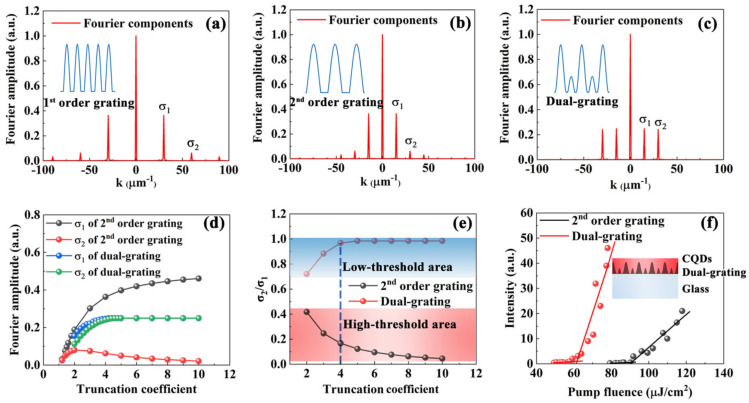
Fourier transforms of (**a**) the first-order grating, (**b**) the second-order grating, and (**c**) the dual-grating for the case where T = 4. The first-order and second-order amplitudes of these Fourier transforms are *σ*_1_ and *σ*_2_, respectively. The insets show the simulated cross-sectional profiles of the grating structures. (**d**) Amplitude of the Fourier transforms of the second-order grating and the dual-grating as a function of T. (**e**) *σ*_2_/*σ*_1_ as a function of the T. (**f**) Thresholds of the microlasers based on the second-order grating and the dual-grating. The inset denotes the schematic of dual-grating laser.

The Fourier transforms of the first-order grating, the second-order grating, and the dual-grating are shown in Figure 4a–c, respectively. The insets show the simulated cross-sectional profiles of the gratings when T = 4. The transforms show that the Fourier order *σ*_2_/*σ*_1_ is very small for the conventional gratings shown in Figure 4a,b, which implies that the lasing threshold in both cases is high. For these conventional gratings, *σ*_1_ and *σ*_2_ are interconnected and cannot be adjusted by varying the grating parameters. For the dual-gratings, however, *σ*_1_ and *σ*_2_ can be controlled independently by adjusting the two gratings within the cavity. For interference lithography applications, the grating structure can be controlled easily by varying the truncation, which is determined by the exposure time.

Figure 4d shows the Fourier coefficients for a conventional grating (taking the second-order grating as an example) and those for a dual-grating as a function of the truncation coefficient. Obviously, the loss *σ*_1_ of the dual-grating is smaller than that of the conventional second-order grating, and the gain *σ*_2_ of the dual-grating is higher than that of the conventional second-order grating. Figure 4e shows the ratio *σ*_2_/*σ*_1_ as a function of the truncation coefficient for these gratings. For the dual-gratings, the ratio *σ*_2_/*σ*_1_ varies within a range from 0.7 to 1, corresponding to the low-threshold area of the laser device. However, the ratio *σ*_2_/*σ*_1_ of the conventional grating varies from 0 to 0.4, corresponding to a high-threshold area of the laser device. It implies that the threshold of the dual-grating laser is very low with arbitrary truncation values, as indicated in Figure 4e. This illustrates the excellent robustness of the proposed dual-gratings, which can be realized via interference lithography. So, for the proposed sine-like, periodic feedback nanostructures, the interference lithography has unique advantages over other fabrication techniques.

Figure 4f shows the thresholds of the second-order grating and dual-grating lasers, which were measured at a specific truncation coefficient (T = 4), as indicated by the blue dashed line shown in Figure 4e. The inset is the schematic of the dual-grating laser. The thresholds of these second-order and dual-grating lasers are 92 μJ/cm^2^ and 61 μJ/cm^2^, respectively. Thus, the threshold of the dual-grating laser is much lower than that of the conventional DFB laser due to the low loss and high gain properties, as shown in Figure 4f.

Generally, the full-wave electromagnetic design can significantly improve the quality and underline the basic physical concepts. However, the main advantages of the proposed analytical model over other design methods are the global optimization ability and the concise physical picture, as shown in Figure 4. We believe that it is a top-down solution.

**Figure 5:**
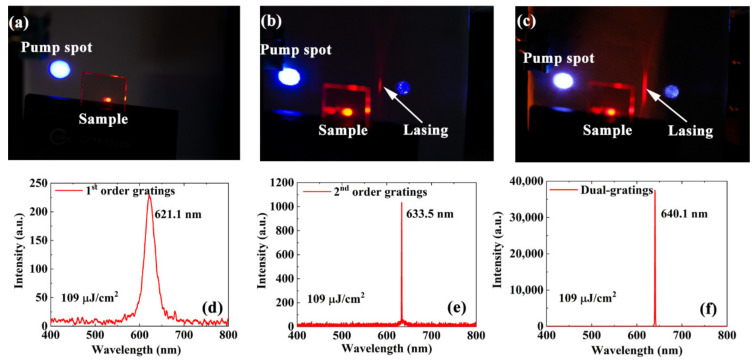
Lasing spots of (**a**) first-order, (**b**) second-order, and (**c**) dual-grating lasers at a pump fluence of 109 μJ/cm^2^. (**d**–**f**) Emission spectra corresponding to the microlasers shown in parts (**a**–**c**), respectively.

Under the same pumping conditions, operating microlasers based on the first-order gratings, the second-order gratings, and the dual-gratings are shown in Figure 5a–c, respectively. The same nanosecond pulse laser is used as the pump source. The pump fluence is fixed at 109 μJ/cm^2^. No lasing spot is observed in the cavity based on the first-order gratings shown in Figure 4a. The lasing spot shown in the dual-grating cavity is much brighter than that shown in the second-order grating cavity. The output energy of the laser based on the dual-grating cavity is approximately 35 times that of the laser based on the second-order grating cavity. The corresponding emission spectra of these lasers are shown in Figure 5d–f, respectively. Only a broad emission spectrum with an FWHM of approximately 45 nm can be obtained when using the first-order gratings, as shown in Figure 5d. The peak wavelength is located at 621.1 nm. There are very sharp lasing peaks with an FWHM of approximately 1 nm for both the second-order gratings and the dual-gratings lasers. The peak wavelengths of the second-order gratings and the dual-grating lasers are located at 633.5 nm and 640.1 nm, respectively. The wavelength shift originates from the differences in the duty cycle of the samples.

**Figure 6:**
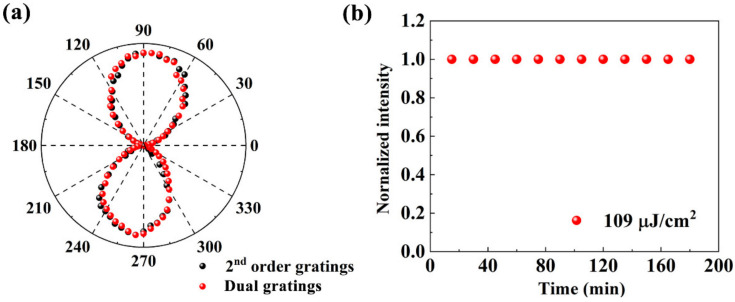
(**a**) Characterization of the polarizations of the second-order DFB laser and the dual-grating laser. (**b**) Stability characterization of the dual-grating lasers.

The polarizations of the traditional second-order DFB laser and the dual-grating lasers were measured, with results shown in Figure 6a. The figure shows that the polarization distribution of the dual-grating laser is the same as that of the second-order DFB laser. The output intensity of the dual-grating laser is shown as a function of time in Figure 6b, where the pump fluence is 109 μJ/cm^2^ at room temperature. No obvious output power attenuation or fluctuations were observed within a period of several hours. Therefore, the laser device shows a high level of stability because of the high stability of the CQDs against photobleaching.

## 4. Conclusions

A high-performance DFB laser based on dual-grating structures is designed in this paper. The dual-grating is formed by integrating a first-order grating with a second-order grating in a PR film and is fabricated via interference lithography. CQDs are used as the gain materials. The PLQY of the CQDs exceeds 85%, and a toluene solution of the CQDs can easily be used to form a film on the dual-gratings via spin coating. The loss and gain characteristics of the cavity can be controlled quantitatively by adjusting the structural parameters of the two dual-grating components. Low-threshold lasing can be achieved through careful balancing of the loss and gain characteristics of the cavity. The high level of stability of the fabricated dual-grating laser can be attributed to the stable properties of the CQDs at room temperature. These results will be helpful in high-performance microlaser design.

## Data Availability

Data is contained within the article.
